# Serum vascular adhesion protein-1 is associated with twelve-year risk of incident cancer, cancer mortality, and all-cause mortality: a community-based cohort study

**DOI:** 10.3389/fonc.2023.1308353

**Published:** 2023-12-13

**Authors:** Szu-Chi Chen, Kang-Chih Fan, I-Weng Yen, Chung-Yi Yang, Chia-Hung Lin, Chih-Yao Hsu, Ya-Pin Lyu, Hsien-Chia Juan, Heng-Huei Lin, Mao-Shin Lin, Shyang-Rong Shih, Hung-Yuan Li, Chun-Heng Kuo

**Affiliations:** ^1^ Division of Endocrinology and Metabolism, Department of Internal Medicine, Taipei City Hospital, Taipei, Taiwan; ^2^ Division of Endocrinology and Metabolism, Department of Internal Medicine, National Taiwan University Hospital, Hsin-Chu, Taiwan; ^3^ Graduate Institute of Clinical Medicine, College of Medicine, National Taiwan University, Taipei, Taiwan; ^4^ Department of Medical Imaging, E-Da Hospital, I-Shou University, Kaohsiung, Taiwan; ^5^ School of Medicine, College of Medicine, I-Shou University, Kaohsiung, Taiwan; ^6^ Department of Obstetrics & Gynecology, National Taiwan University Hospital, Taipei, Taiwan; ^7^ Department of Internal Medicine, College of Medicine, National Taiwan University, Taipei, Taiwan; ^8^ Division of Cardiology, Department of Internal Medicine, National Taiwan University Hospital, Taipei, Taiwan; ^9^ Division of Endocrinology and Metabolism, Department of Internal Medicine, National Taiwan University Hospital, Taipei, Taiwan; ^10^ Center of Anti-Aging and Health Consultation, National Taiwan University Hospital, Taipei, Taiwan; ^11^ School of Medicine, College of Medicine, Fu Jen Catholic University, New Taipei City, Taiwan; ^12^ Department of Internal Medicine, Fu Jen Catholic University Hospital, Fu Jen Catholic University, New Taipei City, Taiwan

**Keywords:** vascular adhesion protein-1, cancer, cancer incidence, cancer mortality, all-cause mortality

## Abstract

**Background:**

Vascular adhesion protein-1 (VAP-1), a dual-function glycoprotein, has been reported to play a crucial role in inflammation and tumor progression. We conducted a community-based cohort study to investigate whether serum VAP-1 could be a potential biomarker for predicting incident cancers and mortality.

**Method:**

From 2006 to 2018, we enrolled 889 cancer-free subjects at baseline. Serum VAP-1 levels were measured using a time-resolved immunofluorometric assay. Cancer and vital status of the participants were obtained by linking records with the computerized cancer registry and death certificates in Taiwan.

**Results:**

During a median follow-up of 11.94 years, 69 subjects developed incident cancers and 66 subjects died, including 29 subjects who died from malignancy. Subjects in the highest tertile of serum VAP-1 had a significantly higher risk of cancer incidence (p=0.0006), cancer mortality (p=0.0001), and all-cause mortality (p=0.0002) than subjects in the other tertiles. The adjusted hazard ratios per one standard deviation increase in serum VAP-1 concentrations were 1.28 for cancer incidence (95% CI=1.01–1.62), 1.60 for cancer mortality (95% CI=1.14–2.23), and 1.38 for all-cause mortality (95% CI=1.09–1.75). The predictive performance of serum VAP-1 was better than that of gender, smoking, body mass index, hypertension, diabetes, and estimated glomerular filtration rate but lower than that of age for cancer incidence, cancer mortality, and all-cause mortality, as evidenced by higher increments in concordance statistics and area under the receiver operating characteristic curve.

**Conclusion:**

Serum VAP-1 levels are associated with a 12-year risk of incident cancer, cancer mortality, and all-cause mortality in a general population.

## Introduction

Based on the Global Cancer Statistics report, the estimated global incidence of cancer for the year 2020 was 19.3 million new cases, while 10.0 million cancer-related deaths occurred worldwide (1). The impact of cancer on global mortality is currently significant, accounting for almost one in six deaths globally. Over the course of the 21^st^ century to date, cancer has surpassed cardiovascular disease as the primary cause of premature death in most countries (2), thereby placing a significant burden on the healthcare system. Implementing preventative measures through the modification of key risk factors presents the most cost-effective long-term strategy for cancer control. Additionally, early detection and timely intervention are crucial factors that can significantly improve the chances of survival for individuals diagnosed with cancer and substantially reduce the financial implications. For this reason, several studies aimed to identify potential biomarkers for the early detection of cancer (3–5).

Inflammation and oxidative stress are important mechanisms involved in many aging-related diseases including cancers, cardiovascular disease, or other diseases associated with disability and mortality (6, 7). Inflammation is recognized as a hallmark feature during the development and progression of cancers. Cytokines, small inflammatory proteins, and infiltrating immune cells derived from tumor and host act in the tumor microenvironment and contribute to the initiation and promotion of carcinogenesis (8). Tumor-derived cytokines and small inflammatory proteins are secreted into systemic circulation and are crucial for the distant metastasis of cancers (8). On the other hand, oxidative stress is recognized as a fundamental process in cancer pathogenesis, contributing to various stages of tumor development and progression, including the transformation of normal cells to tumor cells, tumor proliferation, growth, invasion, angiogenesis, and metastasis (7). Markers of systemic inflammatory and oxidative stress in the circulation could predict cancer progression (9–11). Several studies have reported associations between pre-diagnostic systemic inflammation markers and the risk of developing cancer (12, 13).

Among the various pro-inflammatory proteins, vascular adhesion protein-1 (VAP-1) is notable for its dual functionality. VAP-1 participates in inflammation and is also a source of oxidative stress. As an endothelial adhesion molecule, it contributes to leukocyte rolling, adhesion, and transmigration into sites of infammation (14). Additionally, VAP-1 is known to exhibit semicarbazide-sensitive amine oxidase (SSAO) activity, thereby catalyzing the oxidative deamination of primary amines into aldehydes, hydrogen peroxide, and ammonia. This process generates advanced glycation end products (AGEs) and advanced lipoxidation end products (15). AGEs participate in the pathological mechanisms underlying the development of several types of cancer (16). Several investigations have been conducted in recent years to explore the plausible role of VAP-1 in cancers.

VAP-1 has a soluble form that is detectable in circulation, rendering it a biomarker for various diseases. For example, circulating VAP-1 has been shown to correlate with the risk of cardiovascular events (17, 18), the risk of diabetic complications in humans (19), and chronic liver diseases (20). With respect to cancers, serum VAP-1 level could be used to predict the prognosis of colon cancer (21) and gastric cancer (22, 23). In a previous study, we provided evidence for the potential utility of serum VAP-1 levels as a predictive biomarker for incident cancer (24) and mortality in subjects with type 2 diabetes (25). However, it remains unclear whether circulating VAP-1 could prove useful in predicting cancer incidence and mortality in the general population, as opposed to a specific high-risk population. To address this question, the objective of this study was to investigate whether serum VAP-1 can predict the incidence of cancers, cancer mortality, and all-cause mortality in a community-based cohort study.

## Materials and methods

### Subjects

The study was initiated on 18th December 2007. It was conducted as a prospective cohort study called the Taiwan Lifestyle Study in a community-based setting between 2007 and 2018 (26, 27). We invited residents aged 18 years or older from Yunlin County, Taiwan, to participate in this study, and obtained written informed consent from each participant. The study underwent review and received approval from the Institutional Review Board of National Taiwan University Hospital (approval number: 200706020R). Participants with a history of cancer at baseline were excluded, since the incidence of cancers was one of the outcome measures. Trained nurses administered a questionnaire to obtain data on demographic characteristics, medical history (including a history of cancer), and health-related lifestyle habits of the participants. We also documented the height and weight of each participant to calculate their body mass index (BMI). Blood pressure was measured using a mercury sphygmomanometer with the arm supported at the heart level after the subject had been sitting calmly for 10 min. Three measurements were taken, and the average of the second and third measurements was used for analysis.

A standard 75-g oral glucose tolerance test (OGTT) was conducted following an 8-h overnight fast. An automatic analyzer (Toshiba TBA 120FR, Toshiba Medical Systems Co., Ltd., Tokyo, Japan) was used to measure plasma glucose and high-sensitivity C-reactive protein (hsCRP) concentrations. Plasma concentrations of hemoglobin A1c (HbA1c) were quantified using automatic analyzers (HLC-723 G7 HPLC systems, Tosoh Corporation, Tokyo, Japan) that were certified by the National Glycohemoglobin Standardization Program (NGSP) and standardized to the Diabetes Control and Complications Trial (DCCT) reference assay. The estimated glomerular filtration rate (eGFR) was calculated with the Chronic Kidney Disease Epidemiology Collaboration (CKD-EPI) equation. The serum samples collected for this study were promptly stored at -80°C until the measurement of VAP-1 concentrations was performed. It is noteworthy that serum VAP-1 and its SSAO activity have been previously reported to remain stable for a period of at least 2 years when stored appropriately at -70°C (28). In order to measure serum VAP-1 concentrations, we employed a time-resolved immunofluorometric assay. This involved using a biotin-conjugated monoclonal anti-human VAP-1 antibody(Biotie Therapies Corp., Turku, Finland) as a capturer on a streptavidin-coated microtiter plate. The detection serum VAP-1 bound to the antibody involved the use of a different europium-conjugated anti-human VAP-1 antibody (Biotie Therapies). The resulting time-resolved fluorescence was measured at 615 nm using a fluorometer (Victor^2^ Multilabel Counter, PerkinElmer Finland Oy, Turku, Finland). Serum VAP-1 concentrations were then quantified based on a reference sample of highly purified human serum VAP-1 (Biovian Ltd, Turku, Finland). The standard curves exhibited an R^2^ value of 0.997–1.000. Additionally, quality control samples were used to measure the inter-batch coefficients of variation, which ranged from 3.8 to 10.5%.

### Outcome measures

The main outcome measures of this study were cancer incidence, cancer mortality, and all-cause mortality. To ascertain the outcomes, we established a linkage between the data from the Taiwan Lifestyle Study and the National Registry of Death and Taiwan Cancer Registry database using a unique citizen identifying number. Individual participant identification information was deliberately inaccessible during the analysis. The procedure for linking data and subsequent analysis received approval from the Institutional Review Board of National Taiwan University Hospital (approval number: 201412122RINC). The termination date for the follow-up period was 31th December, 2018. Cancer incidence was defined as the frequency of occurrence of new cancers in the cohort per year. Cancer-free participants were those without a diagnosis of cancer until the end of follow-up. Participants who did not experience mortality, as determined by the National Death Registry at the conclusion of the follow-up period, were classified as survivors. In case of mortality, the underlying cause of death was coded according to the International Classification of Diseases, 9^th^ or 10^th^ Revision, Clinical Modification (ICD-9-CM or ICD-10-CM). Death from cancer was coded if ICD-9 = 140−208 or ICD-10 = C00-C96.

### Statistical analysis

In this study, normally-distributed continuous variables were reported as means and standard deviations (SD) in metric and S.I. units, while continuous variables with skewed distribution were subjected to logarithmic transformation before analysis and reported as medians (interquartile ranges). Categorical variables were expressed as proportions or percentage of patients in the subgroup. To assess the differences in clinical characteristics between subjects who developed cancer and those who did not and between survivors and non-survivors, Student’s *t*-tests and Chi-square tests were performed. Pearson’s correlation coefficients were employed to examine the associations between serum VAP-1 concentrations and clinical characteristics as well as plasma biomarkers. The cumulative incidence of cancer, cancer mortality, and all-cause mortality by tertile of serum VAP-1 concentrations was estimated using Kaplan-Meier survival curves and tested by log-rank tests. The associations between outcomes and serum VAP-1 concentrations were assessed using Cox proportional hazard models. We conducted a multivariable analysis and utilized a stepwise procedure to select potential confounding variables for the incidence of cancer. The full model included the variables age, gender, smoking, body mass index(BMI), hypertension, and diabetes mellitus (DM). These variables were chosen based on their potential impact on the development of cancer and their association with serum VAP-1 concentrations. For cancer mortality and all-cause mortality, we included eGFR as a covariate in the models to adjust for potential confounding effects. We utilized the concordance statistics and the area under the receiver-operating characteristic curve (AUC) to assess the predictive ability of the statistical model for cancer incidence, cancer mortality, and all-cause mortality of the participants during the follow-up period. These metrics were expressed in the range of 0.5 (no predictive ability) to 1 (perfect predictive ability). To determine if a given variable could enhance the predictive ability for the outcomes, we calculated the differences in concordance statistics and AUC with and without the variable. We considered a two-tailed p-value <0.05 to indicate statistical significance. All statistical analyses were performed using Stata/SE 15.0 for Windows (StataCorp LP, College Station, TX).

## Results

This study included 889 subjects enrolled over the period 2006–2010 with a mean age of 62.9 ± 13.9 years. During the median follow-up period of 11.94 years (interquartile range:10.94–12.97 years), a total of 69 subjects developed incident cancer, and 66 subjects died, including 29 subjects who died from malignancy. Among those with incident cancer, the most prevalent diagnoses were breast cancer (n=11), colorectal cancer (n=9), hepatobiliary cancer (n=8), and lung cancer (n=8). Among participants who died from malignancies, the highest proportion was observed for hepatobiliary cancer (n=8), followed by lung cancer (n=5). Individuals who developed cancer and those who died exhibited higher serum VAP-1 concentrations at baseline (**Table 1**). In addition, subjects who developed cancers during follow-up were older, had higher systolic blood pressure, HbA1c levels, lower eGFR, and were more likely to have hypertension and/or DM. Subjects who experienced mortality during the follow-up period were characterized by advanced age, male predominance, higher systolic blood pressure (SBP), fasting plasma glucose (FPG), OGTT 2h plasma glucose (OGTT 2hPG), HbA1c, and hsCRP levels, and lower eGFR. Moreover, a greater proportion of these subjects manifested smoking habits, hypertension, and DM.

**Table 1:** Clinical characteristics in subjects with or without cancer incidence and with or without mortality during follow-up.

	No cancer developed	Cancer developed	p	Alive	Dead	p
N (%)	819	69		823 (92.58)	66 (7.42)	
**Age (years)**	**62.03 ± 13.71**	**73.12 ± 11.64**	**<0.0001**	**61.56 ± 13.25**	**79.67 ± 10.10**	**<0.0001**
**Follow up duration (years)**	**12.12 ± 1.00**	**5.27 ± 3.15**	**<0.0001**	**12.10 ± 1.00**	**7.06 ± 3.24**	**<0.0001**
**Male gender (N, %)**	302 (36.87)	31 (44.93)	0.185	**292 (35.48)**	**42 (63.64)**	**<0.0001**
**Smoking (N, %)**	142(17.36)	16(23.53)	0.134	**88 (10.69)**	**15 (22.73)**	**0.003**
BMI (kg/m^2^)	24.34 ± 3.43	24.10 ± 3.76	0.5775	24.31 ± 3.46	24.47 ± 3.51	0.7314
**SBP (mmHg)**	**124 ± 17**	**128 ± 19**	**0.0422**	**124 ± 17**	**130 ± 18**	**0.0035**
DBP (mmHg)	79 ± 10	79 ± 14	0.7248	79 ± 11	80 ± 10	0.7645
**Hypertension (N, %)**	**239 (29.18)**	**29 (42.03)**	**0.026**	**238 (28.92)**	**30 (45.45)**	**0.005**
**FPG (mg/dL)**	92 ± 19	95 ± 20	0.2139	**92 ± 17**	**99 ± 33**	**0.0029**
**OGTT 2hr glucose (mg/dL)**	125 ± 58	139 ± 69	0.0568	**124 ± 54**	**157 ± 94**	**<0.0001**
**HbA1c (%)**	**5.7 ± 0.8**	**6.0 ± 1.2**	**0.0021**	**5.71 ± 0.74**	**6.18 ± 1.43**	**<0.0001**
**Diabetes mellitus (N, %)**	**88 (10.74)**	**13 (18.84)**	**0.042**	**81 (9.84)**	**20 (30.3)**	**<0.0001**
**Creatinine (mg/dL)**	1 (0.9-1.1)	1.1 (0.9-1.2)	0.0735	**1 (0.9-1.1)**	**1.1(1-1.2)**	**0.0002**
**eGFR (mL/min/1.73m^2^)**	**66.96 ± 11.70**	**60.57 ± 1.31**	**<0.0001**	**67.12± 11.63**	**58.22 ± 10.05**	**<0.0001**
**hsCRP (mg/dL)**	0.09(0.05-0.17)	0.12 (0.05-0.19)	0.3985	**0.09 (0.05-0.17)**	**0.13 (0.05-0.24)**	**0.0061**
**Serum VAP-1 (ng/mL)**	**500.20 ± 142.58**	**582.71 ± 153.14**	**<0.0001**	**498.00 ± 139.19**	**613.88 ± 171.58**	**<0.0001**

Means ± SD or medians (interquartile ranges) are shown. Plasma triglyceride and hsCRP were logarithmically transformed for statistical analyses.

BMI, body mass index; SBP, systolic blood pressure; DBP, diastolic blood pressure; FPG, fasting plasma glucose; OGTT, oral glucose tolerance tests; HbA1C, glycated hemoglobin; eGFR, estimated glomerular filtration rate by the CKD-EPI equation; hsCRP: high sensitivity C-reactive protein; VAP-1, Vascular adhesion protein-1.

The bold values in the tables signify parameters that exhibit statistically significant differences (p-value <0.05).

**Table 2** presents the correlation between serum VAP-1 concentrations and the clinical characteristics and plasma biomarkers in the studied subjects. Serum VAP-1 concentration was positively correlated with age, HbA1C, FPG, OGTT 2h PG, and SBP, and negatively correlated with eGFR. No significant associations were found between serum VAP-1 concentrations and BMI, diastolic blood pressure (DBP), or hsCRP levels.

**Table 2:** Relationship between serum vascular adhesion protein-1 (VAP-1) concentrations and clinical characteristics.

	R^2^	p-value
**Age**	**0.3688**	**<0.0001**
BMI	-0.0493	0.1437
**HbA1c**	**0.2957**	**<0.0001**
**Fasting plasma glucose**	**0.2577**	**<0.0001**
**OGTT 2hr glucose**	**0.3036**	**<0.0001**
**SBP**	**0.1430**	**<0.0001**
DBP	0.0503	0.1346
Log plasma hsCRP	-0.0303	0.3673
**eGFR**	**-0.2615**	**<0.0001**

VAP-1, vascular adhesion protein-1; BMI, body mass index; HbA1C, glycated hemoglobin; OGTT, oral glucose tolerance tests; SBP, systolic blood pressure; DBP, diastolic blood pressure; eGFR, estimated glomerular filtration rate by the CKD-EPI equation; hsCRP: high sensitivity C-reactive protein.

The bold values in the tables signify parameters that exhibit statistically significant differences (p-value <0.05).

**Figure 1:**
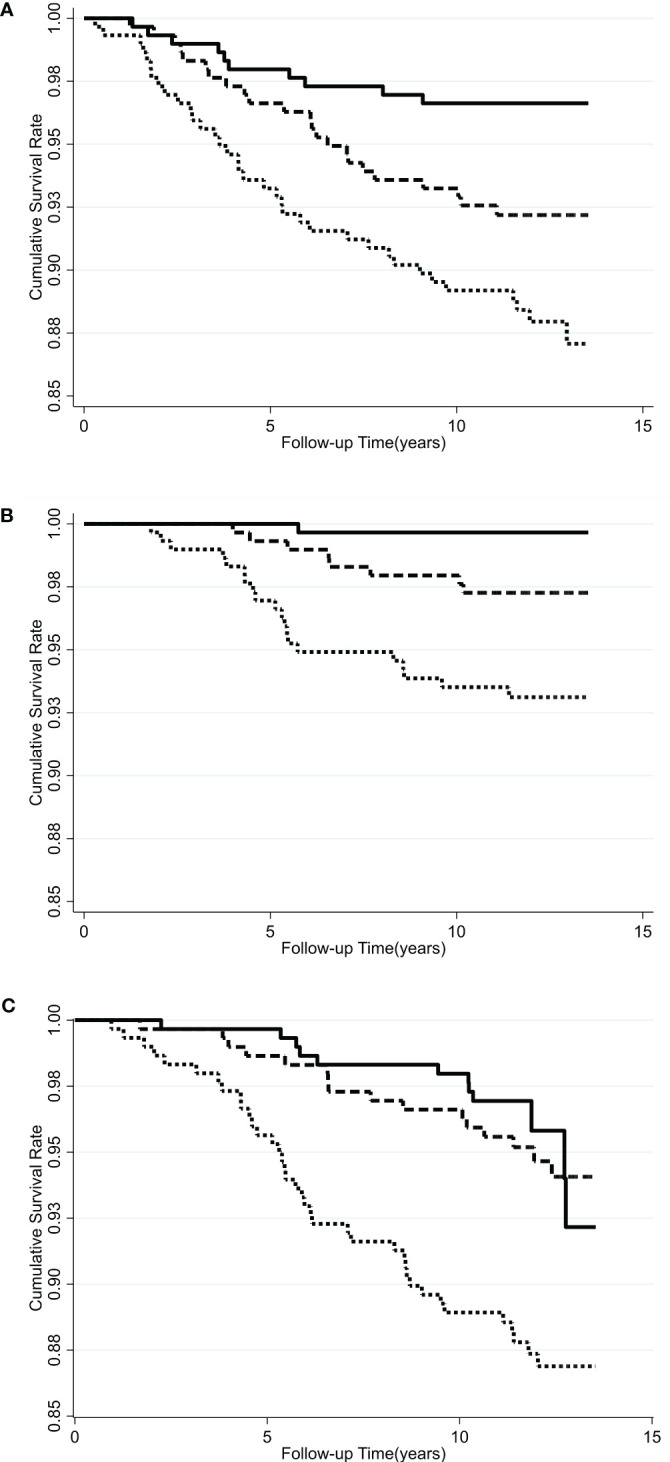
Kaplan-Meier curves for the cumulative incidence of subjects who were free of **(A)** cancers, **(B)** cancer mortality, and **(C)** all-cause mortality among different tertiles of serum VAP-1 concentration. Solid line, lowest tertile of plasma VAP-1 concentration; long dash line, middle tertile of serum VAP-1 concentration; dash line, highest tertile of serum VAP-1 concentration. P =0.0006 by log-rank test for incident cancer, 0.0001 for cancer mortality, and 0.0002 for all-cause mortality.

Kaplan-Meier survival curves indicate that the subjects with the highest serum VAP-1 levels had greater incidence of cancer (p=0.0006), cancer mortality (p=0.0001), and all-cause mortality (p=0.0002) compared to other tertiles during the 11.7-year follow-up (**Figure 1**). The hazard ratios (HRs) of serum VAP-1 for incident cancers, cancer mortality, and all-cause mortality were calculated using Cox proportional hazard models (**Table 3**). In the univariate analysis, elevated levels of serum VAP-1 were associated with an increased risk of incident cancer, cancer mortality, and all-cause mortality. The adjusted HR per 1 SD increase in serum VAP-1 concentrations in the model developed by forward, backward, and stepwise selection was 1.29 for cancer incidence (95% CI = 1.03–1.08), 1.60 for cancer mortality (95% CI = 1.16–2.19) and 1.39 for all-cause mortality (95% CI = 1.12–1.73). In the full model adjusting for all potential confounders, serum VAP-1 levels could significantly predict incident cancer, cancer mortality, and all-cause mortality. The adjusted HR per 1 SD increase in serum VAP-1 concentrations were 1.28 for cancer incidence (95% CI = 1.01–1.62), 1.60 for cancer mortality (95% CI = 1.14–2.23), and 1.38 for all-cause mortality (95% CI = 1.09–1.75).

**Table 3:** Hazard ratios (HRs) (95% confidence intervals, 95% CI) of serum vascular adhesion protein-1 (VAP-1) concentrations in predicting cancer incidence, cancer mortality, and all-cause mortality in unadjusted and adjusted models.

HRs (95% CIs) per 1 SD increase in serum VAP-1 concentrations	Unadjusted	Adjusted model by stepwise model selection	Adjusted model including all potential confounders
Cancer incidence	1.53* **(1.27-1.84)**	**1.29** ^‡^ **(1.03-1.08)**	**1.28** ^‡^ **(1.01-1.62)**
Cancer mortality	1.86* **(1.46-2.38)**	**1.60** ^†^ **(1.16-2.19)**	**1.60** ^†^ **(1.14-2.23)**
All-cause mortality	1.74* (1.46-2.06)	**1.39^†^ (1.12-1.73)**	**1.38^†^ (1.09-1.75)**

One standard deviation (SD) of serum VAP-1 = 144.95 ng/ml.

*p < 0.001, ^†^p < 0.01, ^‡^p < 0.05.

An adjusted model by stepwise model selection: for cancer incidence, adjusted for age; for cancer mortality, adjusted for age; for all-cause mortality, adjusted for age, smoking, and estimated glomerular filtration rate.

An adjusted model including all potential confounders: for cancer incidence, adjusted for age, gender, smoking, body mass index, hypertension, and diabetes; for cancer mortality, adjusted for age, gender, smoking, body mass index, hypertension, diabetes, and estimated glomerular filtration rate; for all-cause mortality, adjusted for age, gender, smoking, body mass index, hypertension, diabetes, and estimated glomerular filtration rate.

The bold values in the tables signify parameters that exhibit statistically significant differences (p-value <0.05).

**Table 4** presents the incremental predictive capacity of distinct variables concerning incident cancer, cancer mortality, and all-cause mortality. In the full model that included all predictors, the concordance statistics and AUC were 0.7157 and 0.7276, respectively, for predicting cancer incidence; 0.8595 and 0.8599, respectively, for predicting cancer mortality; 0.8612 and 0.8818, respectively, for predicting all-cause mortality. For the prediction of incident cancer, the increment in concordance statistics and AUC by serum VAP-1 were 0.004 and 0.0057, respectively, which was higher than that of gender, smoking, BMI, hypertension, and DM but lower than that of age. For the prediction of cancer mortality, the respective increments were 0.0179 and 0.0175, which was higher than that of gender, smoking, BMI, hypertension, DM, and eGFR but lower than that of age. For the prediction of all-cause mortality, the respective increments were 0.0085 and 0.0081, which was higher than that of gender, smoking, BMI, hypertension, DM, and eGFR but lower than that of age. These findings suggest that serum VAP-1 can enhance the prediction of cancer incidence, cancer mortality, and all-cause mortality, and that its performance in improving predictions is superior to that of other predictors, with the exception of age.

**Table 4:** Concordance statistics (C-statistics) and area under the receiver operating characteristic curve (AUC) with and without indicated variables in models predicting cancer incidence, cancer mortality, and all-cause mortality.

	Cancer incidence		Cancer mortality		All-cause mortality	
	C-statistics	AUC	C-statistics	AUC	C-statistics	AUC
Full model	0.7157	0.7276	0.8595	0.8599	0.8612	0.8818
Variable deleted from the model						
Serum VAP-1	0.7117 (0.004)	0.7219 (0.0057)	0.8416 (0.0179)	0.8424 (0.0175)	0.8527 (0.0085)	0.8737 (0.0081)
Age	0.6542 (0.0615)	0.6634 (0.0642)	0.8153 (0.0442)	0.8175 (0.0424)	0.7930 (0.0682)	0.8085 (0.0733)
Gender	0.7168 (-0.0011)	0.7288 (-0.0012)	0.8563 (0.0032)	0.8562 (0.0037)	0.8581 (0.0031)	0.8788 (0.003)
Smoking	0.7168 (-0.0011)	0.7354 (-0.0087)	0.8587 (0.0008)	0.8591 (0.0008)	0.8570 (0.0042)	0.8772 (0.0046)
BMI	0.7202 (-0.0045)	0.7330 (-0.0054)	0.8627 (-0.0032)	0.8623 (-0.0024)	0.8622 (-0.001)	0.8827 (-0.0009)
Hypertension	0.7157 (0)	0.7278 (-0.0002)	0.8571 (0.0024)	0.8584 (0.0015)	0.8615 (-0.0003)	0.8820 (-0.0002)
DM	0.7157 (0)	0.7277 (-0.0001)	0.8586 (0.0009)	0.8588 (0.0011)	0.8614 (-0.0002)	0.8815 (0.0003)
eGFR			0.8613 (-0.0018)	0.8621 (-0.0022)	0.8603 (0.0009)	0.8811 (0.0007)

VAP-1, vascular adhesion protein-1; BMI, body mass index; DM, diabetes mellitus; eGFR, estimated glomerular filtration rate by the CKD-EPI equation.

Differences between the full model and the model without the indicated variable are shown in parentheses.

## Discussion

To the best of our knowledge, this study represents the first report that elevated circulating VAP-1 concentrations can independently predict the risk of incident cancers, cancer-related mortality, and all-cause mortality in a general population. The predictive ability of serum VAP-1 for incident cancer was greater than that of gender, smoking, BMI, hypertension, and DM but lower than that of age. The risk of cancer mortality and all-cause mortality was more reliably predicted by serum VAP-1 than by gender, smoking, BMI, hypertension, DM, and eGFR. Together these results suggest that serum VAP-1 could be a useful biomarker in addition to traditional risk factors to predict incident cancers, cancer mortality, and all-cause mortality.

In the present study, serum VAP-1 emerged as an independent predictor of incident cancer and cancer mortality, even after adjusting for traditional risk factors. The factors of greater age, smoking, and obesity are well established as being significantly associated with an increased risk of cancer development (29). Additionally, individuals with diabetes or hypertension are known to be at higher risk for cancer and cancer mortality (30, 31). Our investigation found a greater increase in concordance statistics and AUC for serum VAP-1 than for gender, smoking, BMI, hypertension, and DM, thereby indicating its superior predictive value in forecasting the occurrence of incident cancer. This finding is supported by previous reports showing the association of VAP-1 with cancer-related events. Individuals diagnosed with hepatocellular cancer exhibited elevated serum VAP-1 levels in comparison to patients with liver cirrhosis alone (20). VAP-1 expression was linked to the progression of tumor invasion and patient survival in breast carcinoma and astrocytoma (32, 33). Among individuals with prostate cancer, serum VAP-1 levels were elevated in those with bone metastases in comparison to those without (34). In addition, previous studies have reported that serum VAP-1 levels were higher in subjects with colorectal cancer compared to healthy volunteers, and that serum VAP-1 levels could serve as an independent prognostic biomarker (21). The collective results of these studies suggest a potential role for VAP-1 in cancer growth and metastasis. However, contrasting results were reported by Toiyama et al., who found that the mean sVAP-1 level was significantly higher in Japanese patients with colorectal cancer than in controls, but the level decreased with disease progression (35). Another study demonstrated that both mean serum VAP-1 levels and tissue VAP-1 protein levels were significantly lower in colorectal cancer patients compared to healthy individuals. However, it is important to note that the sample size of this study was relatively small, consisting of only 31 patients with colorectal cancer and 31 age- and sex-matched controls (36). Comparable results have been reported in individuals with gastric cancer, showing that low sVAP-1 levels were associated with poor prognosis (22, 23). It is worth noting that in our cohort, the number of participants with gastric cancer was limited. Although there may be variations in the relationship between VAP-1 and cancer across different types of cancer, our study provides support for the predictive value of VAP-1 in assessing the risk of cancer incidence and poor prognosis from a general population perspective.

Several potential mechanisms have been proposed to link VAP-1 with cancer progression. VAP-1 has been found to be expressed on tumor vascular endothelium in various types of cancer by immunohistochemical (IHC) staining, such as hepatocellular carcinoma, colorectal cancer, and head and neck cancer, and has also been involved in the recruitment of lymphocytes to cancer vasculature (21, 37, 38). In our previous study, we used IHC analysis to demonstrate a significant upregulation of VAP-1 expression at the invasion front of colorectal cancer compared to its expression in the main tumor. This finding suggests that VAP-1 may play a role in tumor invasion and metastasis (21). Additionally, VAP-1 has been proposed to promote tumor growth by facilitating the recruitment of Gr-1+CD11b+ myeloid cells into tumors, and increase cancer cell extravasation and angiogenesis in melanoma and lymphoma (39, 40). VAP-1 could also regulate IL-1β–stimulated M2 macrophage infiltration and induce lymphangiogenesis and angiogenesis (41). In patients with glioma, VAP-1 expression in tumors was associated with stronger staining of M2 macrophage markers and could be a predictor of a poor prognosis (42). Additionally, VAP-1’s SSAO activity could potentially provide another mechanism that links to cancer progression. SSAO is known to catalyze oxidative deamination reactions that result in the production of hydrogen peroxide, a potent source of oxidative stress, and aldehyde, which is a precursor of AGEs. Both elevated oxidative stress and the interaction between AGE and its receptor have been associated with the development of cancers (43, 44). Studies have revealed that serum SSAO activity was positively correlated with angiogenic factor VEGF in patients with non-small-cell lung cancer (45). SSAO inhibitors were shown to suppress tumor progression and attenuate neo-angiogenesis of hepatocellular tumors in mice (40). T. Kinoshita et al. have demonstrated in murine colon cancer models that VAP-1 has a role in the generation of an immunosuppressive tumor microenvironment through the H_2_O_2_-associated Th2/M2 conditions (46). Intraperitoneal administration of the VAP‐1 inhibitor U‐V296 suppressed tumor growth by enhancing tumor antigen‐specific CD8+ T cells. In addition, they also observed a synergistic anti‐tumor effect of VAP-1 inhibitors in combination with immune checkpoints inhibitors. In oral squamous cell carcinoma, downregulation of VAP-1 suppressed tumor cell proliferation, migration, and invasion *in vitro* and inhibited tumor proliferation and metastasis *in vivo* through reducing NF-κB/IL-8 signaling and decreasing neutrophil infiltration (47). According to these studies, VAP-1 inhibitors may have a role in treating patients with cancers. It is worth noting that VAP-1 inhibitors have already been developed and are currently under clinical trials for the treatment of diabetic retinopathy and diabetic kidney disease in human (48, 49). Findings from the literature and the present study indicate that exploration of VAP-1 inhibitors in treating cancers are promising and should be performed in the future. In addition, all these findings provide potential mechanisms supporting the association between serum VAP-1 and incident cancers as well as cancer mortality observed in the current study.

This study has several strengths including the well-characterized clinical parameters, population-based recruitment flow, and accurate records of the incidence of cancers and vital status with a long-term follow-up. Moreover, the utilization of the time-resolved immunofluorometric assay, which possesses high sensitivity, allowed the detection of subtle differences in serum VAP-1 levels. However, some limitations of our study should be considered. First, since the recruited subjects were limited to Han Chinese people, it is unclear whether the present findings can be generalized to other ethnicities. Second, the number of subjects who developed cancers is relatively small. During the 11.94-years follow-up period, a total of 69 subjects developed incident cancer. The annual cancer incidence rate was 0.65%, which is lower than the 1.106% annual cancer incidence rate observed in subjects with diabetes, as presented in our previous reports (24). The predominant cancer types were breast cancer (n=11), colorectal cancer (n=9), hepatobiliary cancer (n=8), and lung cancer (n=8), which is similar to the epidemiological survey in Taiwan (50). Since the numbers for different types of cancer are also limited, we are unable to perform analyses on the relationship between serum VAP-1 and specific cancer types. Future investigations with larger sample sizes will be required to address this issue and provide more comprehensive insights into the association between VAP-1 and various types of cancer.

## Conclusion

Our study provides evidence of the association between elevated serum VAP-1 levels and an increased risk of cancer incidence, cancer mortality, and all-cause mortality. These findings indicate that serum VAP-1 might constitute a promising novel biomarker for predicting the probability of incident cancer and mortality in the general population. However, additional investigations are required to clarify the underlying mechanisms and potential clinical applications of our findings, which may ultimately aid in the development of more efficacious screening and treatment approaches.

## Data availability statement

The datasets presented in this article are not readily available because the applicability of the data from the National Registry of Death and Taiwan Cancer Registry database is restricted. Requests to access the datasets should be directed to Ministry of Health and Welfare, https://dep.mohw.gov.tw/DOS/cp-5283-63826-113.html.

## Ethics statement

The studies involving humans were approved by the Institutional Review Board at the National Taiwan University Hospital (202210022RINB). The studies were conducted in accordance with the local legislation and institutional requirements. The participants provided their written informed consent to participate in this study.

## Author contributions

SC: Formal Analysis, Methodology, Project administration, Writing – original draft, Conceptualization, Investigation. KF: Data curation, Methodology, Project administration, Writing – review & editing, Formal Analysis, Investigation. IY: Funding acquisition, Methodology, Project administration, Resources, Writing – review & editing, Formal Analysis, Investigation. CY: Investigation, Methodology, Writing – review & editing, Formal Analysis. CL: Data curation, Funding acquisition, Methodology, Project administration, Writing – review & editing, Formal Analysis, Investigation. CH: Methodology, Project administration, Writing – review & editing, Formal Analysis, Investigation. YL: Data curation, Formal Analysis, Writing – review & editing, Investigation, Methodology. HJ: Writing – review & editing, Project administration, Formal Analysis, Data curation, Investigation, Methodology. LH: Data curation, Project administration, Writing – review & editing, Formal Analysis, Investigation, Methodology. ML: Conceptualization, Data curation, Writing – review & editing, Formal Analysis, Investigation, Methodology. SS: Conceptualization, Data curation, Writing – review & editing, Formal Analysis, Investigation, Methodology. HL: Conceptualization, Supervision, Validation, Writing – review & editing, Formal Analysis, Investigation, Methodology. CK: Conceptualization, Methodology, Supervision, Validation, Writing – review & editing, Formal Analysis, Funding acquisition, Investigation, Project administration.
